# Molecular profiling of NOD mouse islets reveals a novel regulator of insulitis onset

**DOI:** 10.1038/s41598-024-65454-x

**Published:** 2024-06-25

**Authors:** Andreas Frøslev Mathisen, Andrei Mircea Vacaru, Lucas Unger, Elena Mirela Lamba, Oana-Ana-Maria Mardare, Laura Maria Daian, Luiza Ghila, Ana-Maria Vacaru, Simona Chera

**Affiliations:** 1 Department of Clinical Science, Mohn Research Center for Diabetes Precision Medicine, University of Bergen, Bergen, Norway; 2 BetaUpreg Research Group, Institute of Cellular Biology and Pathology “Nicolae Simionescu”, Bucharest, Romania

**Keywords:** Mechanisms of disease, Transcriptomics, Pre-diabetes, Induced pluripotent stem cells

## Abstract

Non-obese diabetes (NOD) mice are an established, spontaneous model of type 1 diabetes in which diabetes develops through insulitis. Using next-generation sequencing, coupled with pathway analysis, the molecular fingerprint of early insulitis was mapped in a cohort of mice ranging from 4 to 12 weeks of age. The resulting dynamic timeline revealed an initial decrease in proliferative capacity followed by the emergence of an inflammatory signature between 6 and 8 weeks that increased to a regulatory plateau between 10 and 12 weeks. The inflammatory signature is identified by the activation of central immunogenic factors such as *Infg, Il1b*, and *Tnfa*, and activation of canonical inflammatory signaling. Analysis of the regulatory landscape revealed the transcription factor *Atf3* as a potential novel modulator of inflammatory signaling in the NOD islets. Furthermore, the Hedgehog signaling pathway correlated with *Atf3* regulation, suggesting that the two play a role in regulating islet inflammation; however, further studies are needed to establish the nature of this connection.

## Introduction

Diabetes is defined by persistent hyperglycemia caused by impaired production or sensing of insulin. Insulin is mainly produced in the endocrine pancreas, by the beta cells. Together with other endocrine cell types involved in glycemia control, such as the glucagon-secreting alpha cells and somatostatin-secreting delta cells, these cells form the pancreatic islets. The two most prevalent diabetes types (Type 1 and Type 2 respectively) are complex diseases resulting from intricate interactions between a composite genetic component and multiple environmental factors. Type 1 diabetes (T1D) is an autoimmune disorder in which insulin-secreting beta cells are gradually targeted and destroyed by the patient’s immune system^1^. The resulting total or near-total beta-cell mass loss leads to a steep decrease in insulin levels and consequent chronic hyperglycemia. When left untreated, the condition leads to death, as the T1D patients require constant exogenous insulin administration to regulate their glycemia^2^. Despite this lifesaving treatment, there is a constant risk of complications, due to imperfect glycemic control. Thus, in the past two decades, considerable effort has been invested in understanding the causal factors of immune attack and developing strategies aimed at beta-cell mass replacement^3,4^.

As a consequence of these sustained efforts, a large repertoire of model systems of different diabetic diseases, as well as beta-cell loss, were developed^5–14^, including in vivo mammalian setups^15–17^. Yet, in contrast with other diabetes forms, reliable model systems for T1D are sparse^18^, with NOD (Non-Obese Diabetes) being the most widely-used animal model for T1D^19^. In NOD mice, diabetes develops spontaneously and is caused by insulitis, i.e., leukocytic infiltration of the pancreatic islets. The incidence of disease onset in NOD mice is impacted by sex, with more females (86%) than males (48%) developing spontaneous diabetes by 30 weeks of age. Moreover, the onset of diabetes occurs earlier in females, with a median incidence at 18 weeks, although a marked decrease in insulin content is already observed at 12 weeks of age^20^. The onset of diabetes is characterized by mild glycosuria (i.e., glucose in urine) and non-fasting hyperglycemia, with customary monitoring starting at approximately 10 weeks of age.

Due to the spontaneous occurrence of diabetes in this strain and its polygenic nature, the exact molecular mechanisms triggering diabetes onset have not been clearly defined. Moreover, the global dynamic transcriptional fingerprint of the timeline leading to diabetes in this model has been understudied. Indeed, probably the most comprehensive and elegant studies mapping the dynamic transcriptional changes characterizing NOD diabetes development, including events preceding diabetes onset, are likely more than a decade old and based on an admittedly old high-throughput technique (microarrays^21^). Newer studies using current technologies have focused mostly on a specific early period, such as the first month after birth^22^, or exclusively on immune cells^23^. Thus, with the current prodigious advances in next-generation sequencing techniques and pathway analysis, there is a current need to molecularly revisit the series of events leading to diabetes onset in NOD mice.

To bridge this gap, we characterized here the events leading to diabetes onset in NOD mice by using next-generation sequencing (RNAseq) combined with pathway analysis on a timeline ranging from 4- to 12-week-old animals (spaced every 2 weeks). We observed a clear emergence of a complex immune system and inflammation signature between 6- and 8 weeks of age, characterized by an increase in key immunogenic factors. Moreover, we pinpointed a transcriptional regulator known to regulate the expression of these genes in the context of other islet stressor conditions (such as hypoxia), which was previously associated with the diabetic NOD phenotype more as a target of the inflammatory milieu, and a driver of beta cell apoptosis^24^. Finally, we explored the connection between this immunogenic factor regulator and the Hedgehog signaling pathway in a pilot experiment.

## Results

### NOD mice undergo changes in islet architecture and exhibit an age-related decrease in the main islet hormones as well as beta- and alpha-cell-specific markers

**Figure 1:**
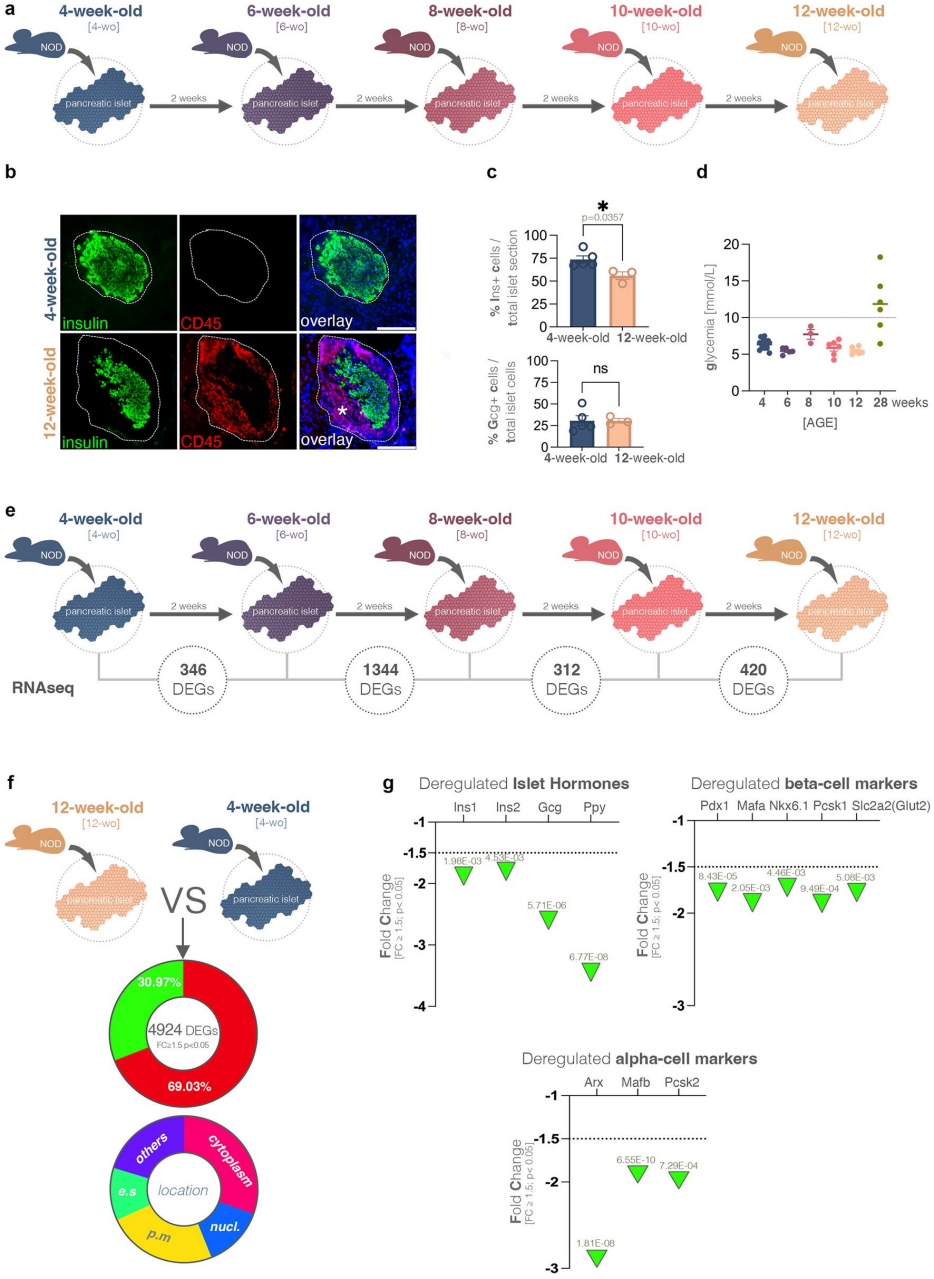
Cellular and molecular changes in NOD mice between 4 and 12 weeks of age. (**a**) Schematic representation of the NOD analysis timeline including the five distinct collection timepoints. (**b**) Representative immunofluorescence images of insulin (green), CD45 (red) and DAPI staining (blue) at 4-weeks and 12-weeks of age in NOD mice (scale 100 µm). (**c**) Graphs depicting the percentage of insulin positive cells (upper panel) or glucagon positive cells (lower panel) per islet section in 4-week-old and 12-week-old NOD mice (non-parametric Mann–Whitney test, each data point (n) represents one mouse, an average of n = 30 islets were counted per animal, error bars: SEM) (**d**) Graph depicting the glycemia values recorder for each analysis timepoint (each data point (n) represents one mouse, error bars: SEM). (**e**) Schematic representation of the five points of comparison and the number of differentially expressed genes (DEGs, FC ≥ 1.5, *p* < 0.05) filtered for each interval. (**f**) Pie diagrams of genes exhibiting significant down- and upregulation (FC ≥ 1.5, *p* < 0.05) as well as their cellular localization in 12-week-old as compared to 4-week-old NOD mice. (**g**) Graphs displaying the observed statistically significant downregulation of islet hormones as well as key beta- and alpha-cells markers in the RNAseq dataset (FC ≥ 1.5, *p* < 0.05).

To comprehensively characterize the early cellular and molecular events leading to diabetes onset in the NOD mice, we analyzed islets from NOD females at five distinct time-points, ranging between 4- and 12 weeks of age (Fig. 1a).

As expected, at 12 weeks of age, the mice exhibited clear signs of insulitis (Fig. 1b, Supp.Fig. 1a asterisks) and changes in islet architecture. The beta-cell population was decreased in the 12-week-old mice compared to the 4-week-old mice (*p* = 0.0357), while the glucagon population remained apparently unchanged (Fig. 1c, green-insulin, red-glucagon, blue-DAPI). Notably, at 12 weeks, all the mice were normoglycemic indicating that they still had the ability to compensate for beta-cell loss (Fig. 1d). In contrast, at 28 weeks, an expected 70% of the mice were hyperglycemic (Fig. 1d, olive).

To analyze the evolution of the islet transcriptional profile in NOD mice, we performed high-throughput sequencing and filtered the differentially expressed genes (DEGs, FC ≤ 1.5,* p* < 0.05, Supplemental Table 1, Supp.Fig. 1b) between the five above-mentioned timepoints (Fig. 1e).

A comparison of the two age extremes (i.e., 12- and 4-week-old) revealed 4924 DEGs, approximately two-thirds (69.03%) of which were upregulated in the mature (12-week-old) animals and had a heterogenous cell compartment distribution (Fig. 1f). Interestingly, except for *Sst*, the main islet hormones were downregulated in 12-week-old NOD mice as compared to their younger counterparts (4 weeks old), a trend also followed by key beta-cell (such as *Pdx1, Mafa, Nkx6.1* amongst others) and alpha-cell (*Arx, Mafb, Pcsk2*) markers (Fig. 1g).

Overall, these data indicate that progressive age-specific alterations of islet architecture and functionality in NOD mice occur between 4 and 12 weeks of age. Thus, to further investigate the progression of this phenotype, we performed pathway analysis of the DEGs characterizing each age interval.

### A steep decrease in proliferative potential is observed between 4 and 6 weeks of age in NOD mice

**Figure 2:**
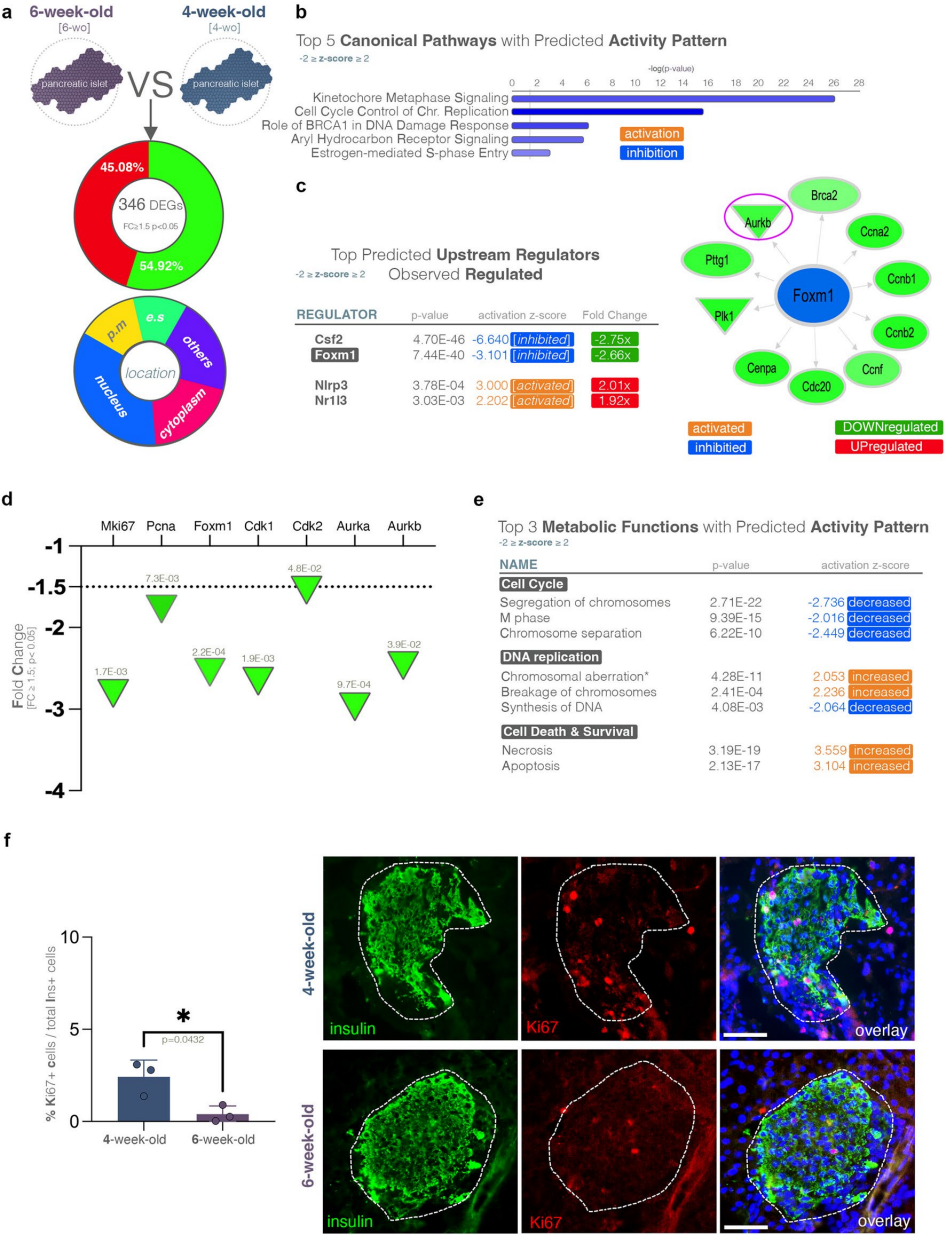
Transcriptional changes in NOD mice between 6-weeks and 4-weeks of age. (**a**) Pie diagrams of genes exhibiting significant down- and upregulation (FC ≥ 1.5, *p* < 0.05) as well as their cellular localization in 6-week-old as compared to 4-week-old NOD mice. (**b**) Top 5 canonical pathways with predicted activation pattern (z-score ≥ 2—activation—orange; z-score ≤ -2—inhibition—blue). (**c**) Top predicted upstream regulators exhibiting observed differential expression (z-score ≥ 2—activated—orange; z-score ≤ -2—inhibited—blue; green -downregulated; red—upregulated). (**d**) Graph displaying the observed statistically significant downregulation of key proliferation markers in the RNAseq dataset (FC ≥ 1.5, *p* < 0.05) between 6 and 4-weeks of age in NOD mice. (**e**) Top 3 metabolic functions with predicted activation pattern (z-score ≥ 2—increased—orange; z-score ≤ -2—decreased—blue). (**f**) Graph depicting the percentage of Ki67 (Mki67) positive cells per islet section in 4-week-old and 6-week-old NOD mice and representative immunofluorescence images (unpaired t-test with Welch’s correction, each data point represents one distinct animal, an average of n = 21 islets/mouse were assessed; error bars: SD; scale—50 µm; green—insulin, red- Ki67, blue—DAPI).

Between 4 and 6 weeks of age, we identified 346 DEGs with no bias toward a certain regulatory pattern (45.08% upregulated, 54.92% downregulated). By cellular location, the largest DEG fraction was categorized as nuclear (Fig. 2a). Pathway analysis revealed that the Top 5 canonical pathways with a predicted activity pattern (− 2 ≥ z-score ≥ 2) were involved in cell cycle progression, and all the pathways were predicted to be inhibited (Fig. 2b). Moreover, *Foxm1*, a key cell cycle regulator, was identified in the top predicted upstream regulators based on the analyzed transcriptional landscape and inferred to be inhibited (Fig. 2c), a prediction confirmed by its observed downregulation in our DEGs set (-2.66x). Similarly, other critical proliferation markers directly regulated by *Foxm1*, such as *Mki67(Ki67)*, *Pcna, Plk1, Cdk1, Cdk2, Aurka,* and *Aurkb,* were also observed to follow the same trend, and were downregulated in 6-week-old NOD mice (Fig. 2d, Supp. Fig. 1c). In addition, the “Diseases and Functions” analysis revealed Cell Cycle, DNA-replication and Cell Death and Survival were the top metabolic processes with predicted activity pattern (Fig. 2e).

These data were confirmed by the quantification of the Ki67-positive beta-cells using immunofluorescence, revealing a steep, significant reduction in the beta-cell proliferative capacity in 6-week-old NOD mice as compared with their young 4-week-old counterparts (Fig. 2f, p = 0.0432, green-insulin, red-Ki67, blue-DAPI).

Taken together, these results suggest a decrease in the islet proliferation potential between 4- and 6-week of age in the NOD mice.

### A strong inflammation signature emerges between 6- and 8-weeks in the islets of NOD mice

**Figure 3:**
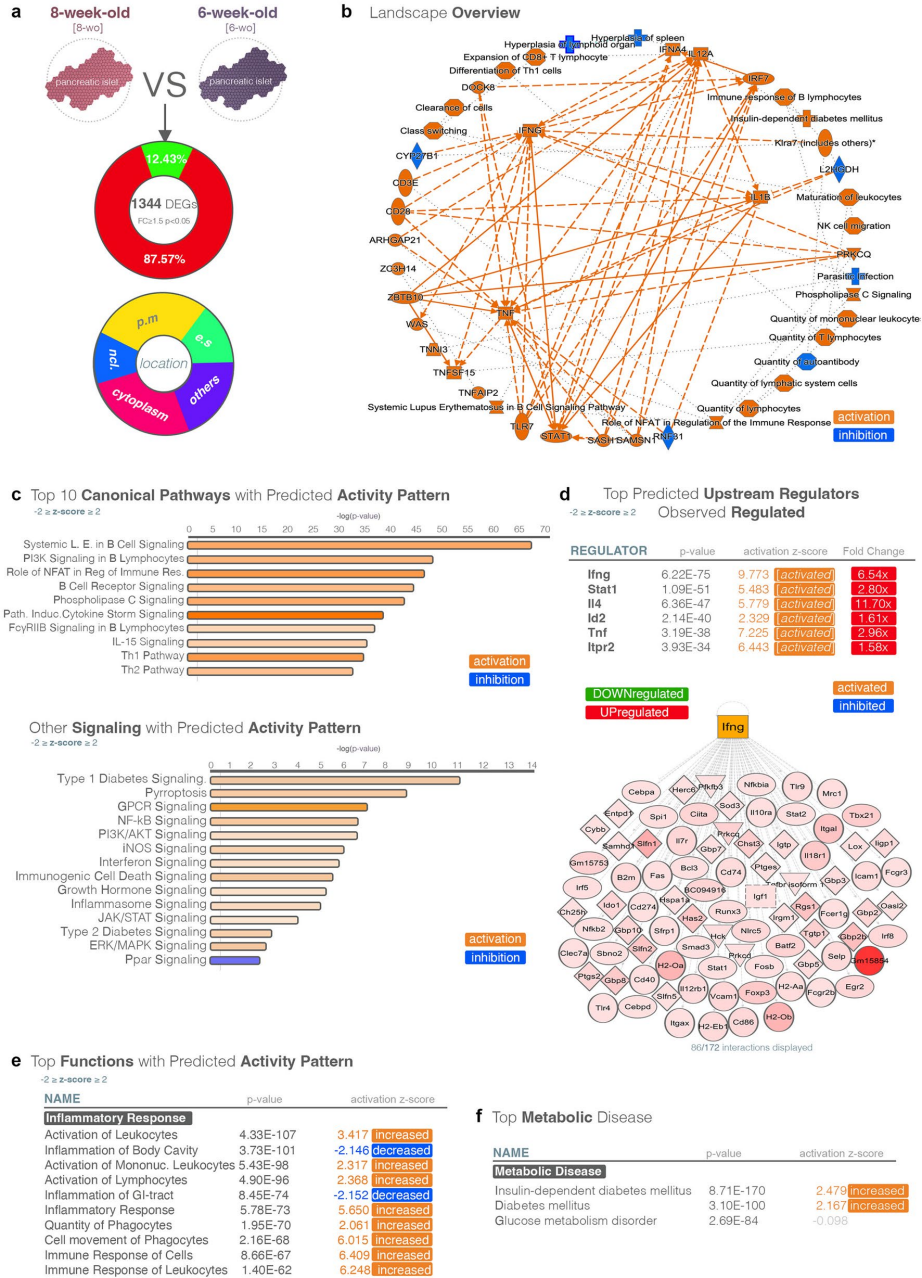
Transcriptional changes in NOD mice between 8-weeks and 6-weeks of age. (**a**) Pie diagrams of genes exhibiting significant down- and upregulation (FC ≥ 1.5, *p* < 0.05) as well as their cellular localization in 8-week-old as compared to 6-week-old NOD mice. (**b**) Regulatory landscape overview characterizing the analyzed transcriptional landscape as generated by pathway analysis. (**c**) Top canonical pathways with predicted activation pattern (z-score ≥ 2—activation—orange; z-score ≤ -2—inhibition—blue). (**d**) Top predicted upstream regulators exhibiting observed differential expression (z-score ≥ 2—activated—orange; z-score ≤ -2—inhibited—blue; green—downregulated; red—upregulated). (**e**) Top functions with predicted activation pattern (z-score ≥ 2—increased—orange; z-score ≤ -2—decreased—blue). (**f**) Top metabolic disease (increased—orange) characterizing the analyzed transcriptional landscape.

The next age interval (6 to 8 weeks) was characterized by the largest DEG set (1344 DEGs) of all analyzed periods (Figs. 1e, 3a), indicating a strong shift in the transcriptional landscape, with the vast majority being upregulated (87.57%). Considering the cellular compartment, most differentially expressed genes were located in the cytoplasm or plasma membrane, suggesting a surge in signaling. Pathway analysis of the transcriptional landscape revealed processes and regulators connected to inflammation and the immune response, with the key immunogenic molecules *Ifng* (interferon gamma), *Il1b* (interleukin 1b) and *Tnf* (tumor necrosis factor) serving as organic centers of the landscape (Fig. 3b). Accordingly, the Top 10 pathways with predicted activity patterns were exclusively involved in key signaling pathways transducing the immune response and inflammation, which were inferred to be activated (Fig. 3c). In addition, other pathways relevant to islet functionality, such as Type 1 Diabetes Signaling, PI3K/AKT Signaling, Ppar Signaling, were identified, and predicted to be activated (Fig. 3c).

Consistently, *Ifng* was the top upstream regulator of the analyzed transcriptional landscape and was strongly inferred to be activated (z-score 9.773), a prediction confirmed by its observed upregulation in our DEGs set (6.54x) (Fig. 3d).

Moreover, the “Diseases and Functions” analysis, revealed processes related to inflammation in the top (Fig. 3e), with “Insulin-dependent diabetes mellitus”, “Diabetes mellitus” and “Glucose metabolism disorders” as top 3 inferred activated metabolic diseases based on the analyzed DEGs landscape (Fig. 3f). Overall, these data suggest the establishment of an immune response signature in NOD mice between 6 and 8 weeks of age.

### Once established, the inflammatory and immune response signatures are maintained or further amplified

**Figure 4:**
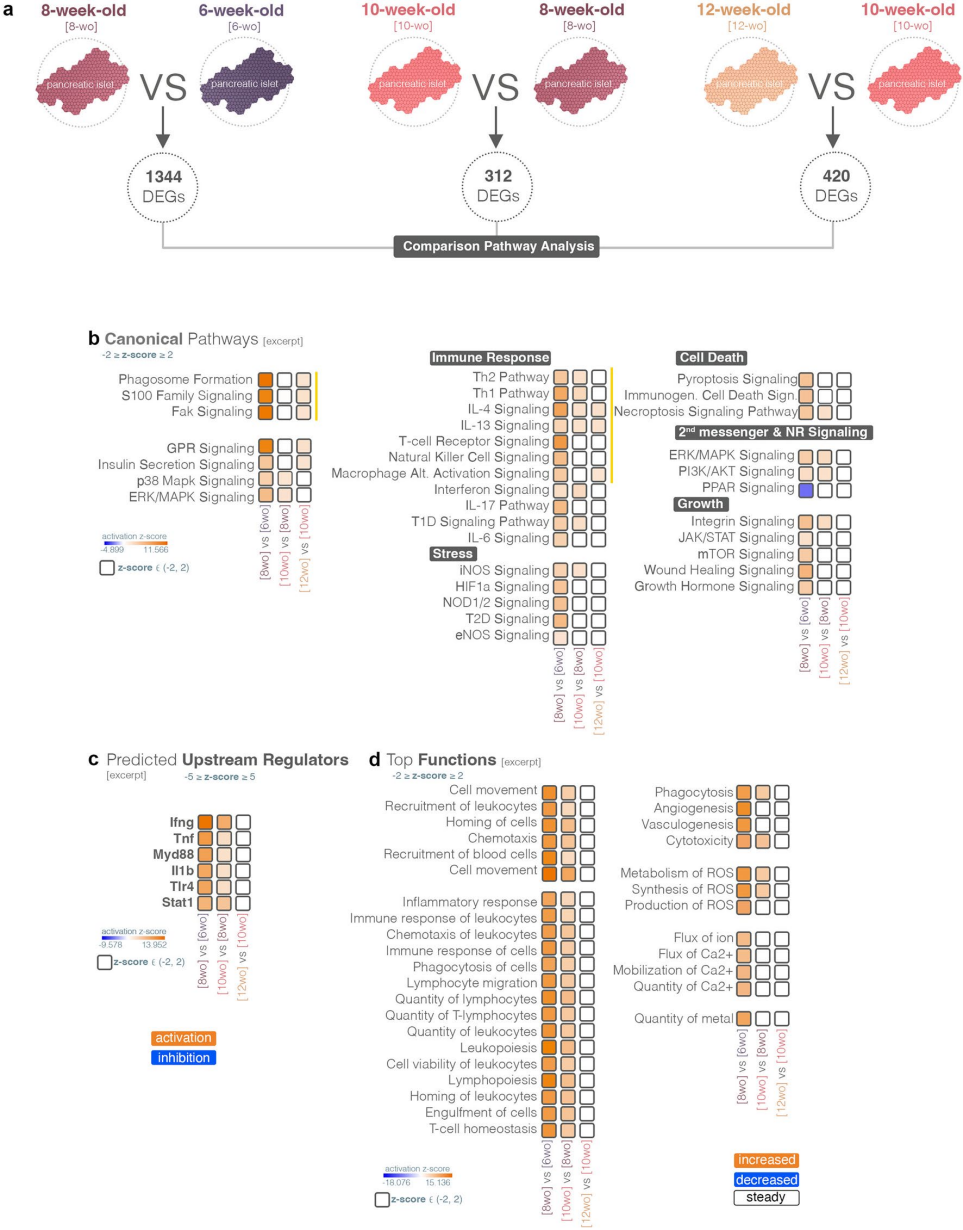
Pathway comparison analysis of the age intervals following inflammation emergence. (**a**) Scheme illustrating the time intervals compared. (**b**) Comparison analysis of the top canonical pathways activity patterns between the different stages compared (z-score ≥ 2—activation—orange; z-score ≤ -2—inhibition—blue). (**c**) Comparison analysis of the top predicted upstream regulators activity patterns between the different stages compared (z-score ≥ 2—activated—orange; z-score ≤ -2—inhibited—blue). (**d**) Comparison analysis of the top functions activity patterns between the different stages compared (z-score ≥ 2—increased—orange; z-score ≤ -2—decreased—blue).

To further analyze the transcriptional landscape regulation over time we performed comparison pathway analysis of the remaining 3 DEGs groups (i.e., DEG^8-weeks vs. 6-weeks^, DEG^10-weeks vs. 8-weeks^ and DEG^12-weeks vs. 10-weeks^, Fig. 4a). This analysis revealed that for some canonical pathways and immune response-, stress-, cell death-, and growth-related signaling, the activation pattern established between 6 and 8 weeks of age was retained, however, it was not amplified nor reversed at later stages (Fig. 4b). In contrast, key immune pathways such as the Th1 and Th2 Pathways as well as Interferon Signaling and T1D Signaling Pathway, displayed reinforced activation during the next period, i.e., between 8 and 10 weeks of age. Similarly, certain cytokine signaling (such as *IL-4* and *IL-13*) was augmented during all the time intervals analyzed.

As expected, the top predicted upstream regulators (Fig. 4c) and functions (Fig. 4d) were related to the immune response, which was inferred to increase during 6 and 8 weeks of age and further amplified in the next period (8 to 10 weeks), to reach a steady state (i.e., maintain the activity state achieved at 10 weeks) in the last analyzed period (10- to 12-weeks).

Overall, the transcriptional timeline indicated an initial decrease in the islet proliferative capacity of the NOD mice following the first month of life, which was immediately succeeded by the establishment of inflammatory and immune response signatures, which were further amplified and maintained in the following stages. However, the molecular mechanism responsible for the initiation of the events leading to islet infiltration, decay and diabetes is still unclear.

### Atf3 and core immunogenic factors are upregulated in NOD islets

**Figure 5:**
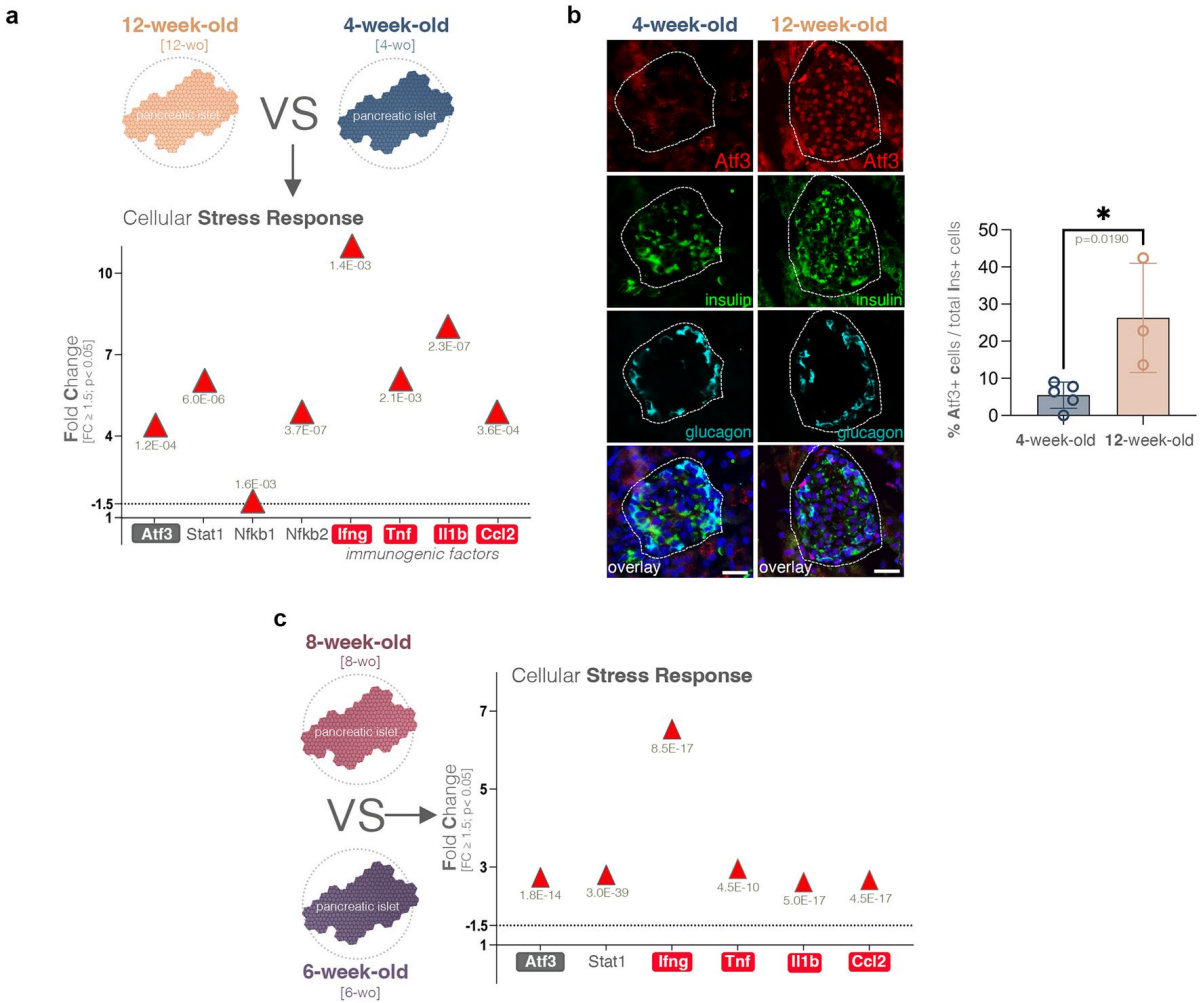
Markers and regulators of the cellular stress response. (**a**) Graph displaying the observed statistically significant upregulation of key cellular stress response markers in the RNAseq dataset (FC ≥ 1.5, *p* < 0.05) in 12-week-old as compared to 4-week-old NOD mice. (**b**) Representative immunofluorescence images and graph depicting the percentage of Atf3 positive cells per islet section in 4-week-old and 12-week-old NOD mice (unpaired t-test with Welch’s correction, each data point represents one distinct animal, an average of n = 18 islets/mouse were assessed; error bars: SD; scale—50 µm; green—insulin, cyan—glucagon red—Atf3, blue—DAPI). (**c**) Graph displaying the observed statistically significant upregulation of key cellular stress response markers in the RNAseq dataset (FC ≥ 1.5, *p* < 0.05) in 8-week-old as compared to 6-week-old NOD mice.

As the inflammatory signature characterizing the NOD timeline predicted the central role of immunogenic factors (Figs. 3b,d, 4c), we examined their presence in the DEG sets. In accordance with the pathway analysis prediction, we found that four key canonical immunogenic factors (*Ifng, Tnf, Il1b* and *Ccl2*) were significantly upregulated in the 12-week-old NOD mice, when compared to their 4-week-old counterparts (Fig. 5a). Moreover, these factors were upregulated as early as the initiation of the immune signature between 6 and 8 weeks of age (Fig. 5c), suggesting their involvement in the early stages of the immune response.

To understand what initiated the immunogenic factors upregulation, we further datamined for potential transcription factors known to directly regulate these molecules. This search identified *Atf3*, a transcription factor that was previously shown to upregulate the expression of *IL-1b, Il-6, Tnfa* and *Ccl2* in the pancreatic islets by being involved in a positive pro-inflammatory loop in response to stress from hypoxia and transplantation^25^. Similar to this previous study, the *Atf3* upregulation in NOD islets mirrored the upregulation of the immunogenic factors (Fig. 5a). Moreover, a significant increase in the number of Atf3 positive (Atf3+) cells (*p* = 0.0190) was revealed by immunofluorescence (Fig. 5b).

Of note, Atf3 was observed upregulated as early as the immune signature establishment between 6- and 8-weeks (Fig. 5c) suggesting its involvement in the early phases of the immune response. Furthermore, other factors implicated in signaling cascades with impact on immunogenic factors modulations (such as *Stat1* and *Nfkb*) followed the same regulatory trend (Fig. 5a,c).

Overall, these data suggest that an Atf3 pro-inflammatory loop promoting core immunogenic factors is involved in the context of NOD islet stress. However, it is unclear which signaling pathways are involved in regulation of *Atf3* expression levels and consequently initiate the pro-inflammatory loop leading, in time, to immune infiltration.

### The Hedgehog signaling pathway exhibits a pattern correlated with Atf3 regulation

**Figure 6:**
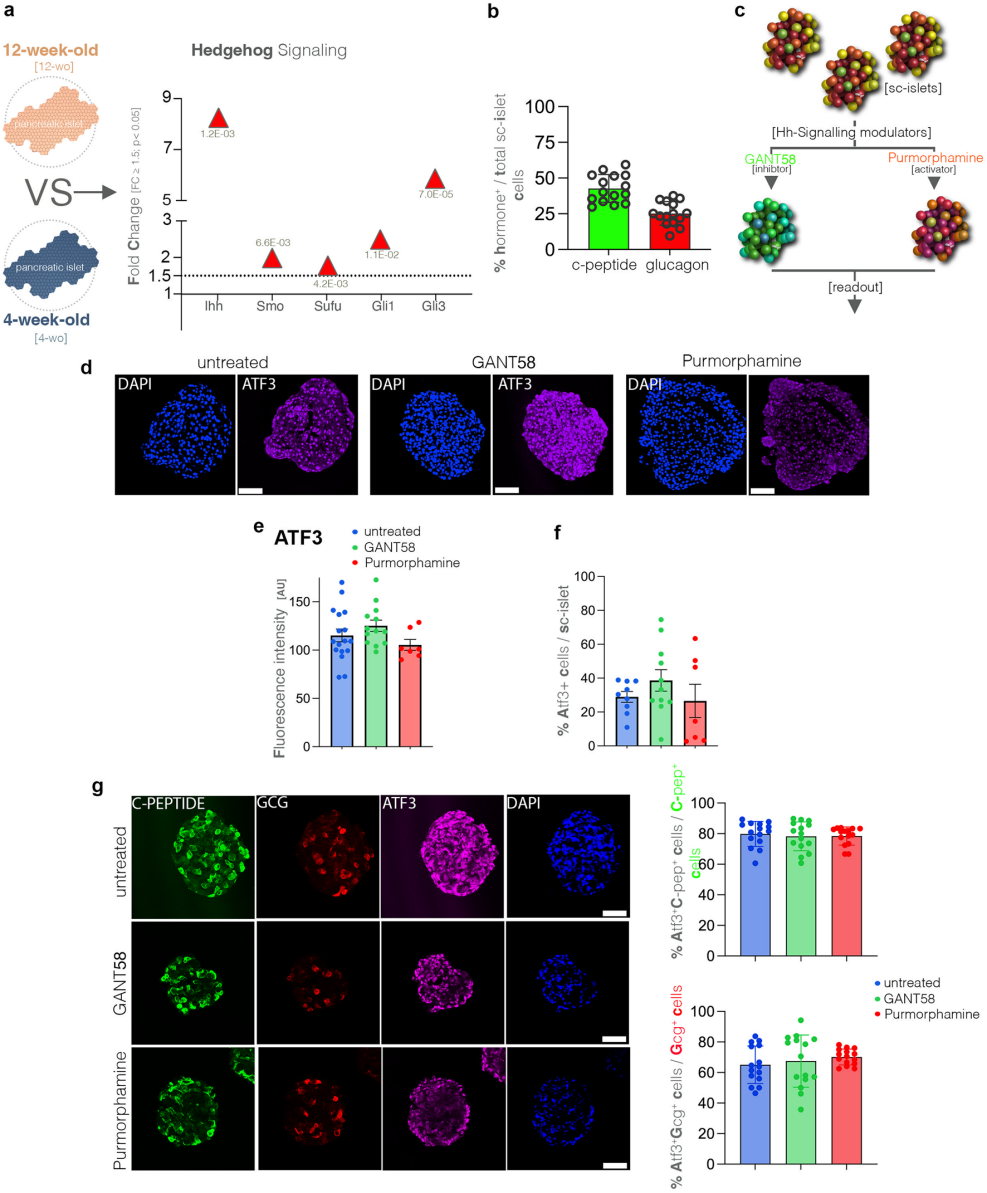
Modulation of the Hedgehog signaling pathway and the cellular stress response. (**a**) Graph displaying the observed statistically significant upregulation of key Hedgehog signaling pathway molecules in the RNAseq dataset (FC ≥ 1.5, *p* < 0.05) in 12-week-old as compared to 4-week-old NOD mice. (**b**) Graph indicating the percentage of Insulin (c-peptide) and Glucagon positive cells from the total sc-islet cells (DAPI) in untreated sc-islets, each data point represents one sc-islet (n = 15), error bars: SD; (**c**) Scheme displaying the experimental setup for modulating the Hedgehog signaling pathway using the Gli-inhibitor GANT58 and the Smo-activator Purmorphamine on human sc-islets. (**d**) Representative immunofluorescence images of ATF3 (purple) and DAPI (blue) staining of untreated, GANT58 and Purmorphamine treated sc-islets (scale bar: 50 µm). (**e**) Graph depicting the ATF3 fluorescence intensity in untreated, GANT58 and Purmorphamine treated sc-islets (non-parametric Mann–Whitney test, each data point (n) represents one sc-islet, error bars: SEM). (**f**) Graph indicating the percentage of ATF3 positive cells per sc-islet (left panel) in untreated, GANT58 and Purmorphamine treated sc-islets (non-parametric Mann–Whitney test, each data point (n) represents one sc-islet, error bars: SEM); (**g**) Representative immunofluorescence images of C-peptide (green), GCG (red), ATF3 (purple) and DAPI (blue) staining of untreated, GANT58 and Purmorphamin treated sc-islets (scale bar: 50 µm) as well as graphs indicating the ratio of ATF3 positive cells from insulin (right panel) and glucagon cells (left panel) for each condition (non-parametric Mann–Whitney test, each data point (n) represents one sc-islet, error bars: SEM).

To identify signaling involved in the regulation of Atf3, we further investigated the largest DEG sets for canonical pathways displaying a regulation similar to the Atf3 immunogenic loop, and uncovered Hedgehog signaling (Hh-signaling), as a potential candidate. Core genes of the pathway such as the ligand *Ihh* (Indian Hedgehog), the receptor *Smo* (Smoothened), critical kinase *Sufu* (Suppressor of fused) and the main transcription factor effectors Gli1 and Gli3 displayed significant upregulation in the 12-week-old NOD mice (Fig. 6a).

To probe a potential connection between the Hh-signaling pathway and Atf3 activation, we performed a pilot experiment in vitro, using human induced pluripotent stem cell-derived islets^26^ (sc-islets) generated by using a recently published protocol by Otonkoski and colleagues^27^. With this very efficient protocol all sc-islets (100%) are expressing both insulin and glucagon, although the ratio of insulin- and glucagon-expressing cells may vary between the islet-like clusters, as indicated by c-peptide (marker for insulin-expressing cells, ≈ 42% ± 9.7) and glucagon (≈ 25.27% ± 8.2) immunofluorescence (Fig. 6b).

An advantage, but also a limitation of this setup, is the fact that analysis is restricted to the islet compartment, and thus it will determine only the potential role of the islet cells in the early events leading to islet infiltration. Along the same lines, it will not allow conclusions about a potential Hh-signaling and ATF3 regulatory loop in the immune cells, nor can identify a potential inter-cellular crosstalk between the islet and immune cells.

In this pilot experiment, the sc-islets were exposed to either a Hh-signaling inhibitor (Gant58, a Gli inhibitor^28^), or a Hh-signaling activator (Purmorphamine, which directly binds and activates Smoothened^29^), followed by the immunofluorescence assessment of the ratio of ATF3+ cells using automated or manual counting (Fig. 6c). This revealed a tendency, albeit not significant, towards lower intensity of ATF3 signal in the sc-islets treated with the Purmorphamine (Fig. 6d,e). Moreover, the ATF3 + ratio from total sc-islet cells (Fig. 6f) as well as insulin-expressing (Fig. 6g) and glucagon-expressing cells (Fig. 6g) was not significantly changed, suggesting that Hh-signaling modulation does not significantly impact ATF3 levels.

## Discussions

Here we used next generation sequencing and pathway analysis to dynamically characterize the global early molecular events defining the pancreatic islets of NOD mice during its progression toward insulitis and diabetes. By comparing the analyzed age extremes, we observed a decline of islet hormones and islet cell signatures between 4 and 12 weeks of age, which was also confirmed for insulin by immunofluorescence quantification. As expected, these changes were doubled by increased insulitis in the 12-week-old islets.

We showed that point-by-point dynamic analysis revealed a decrease in islet proliferative capacity as the first age-related event (between 4 and 6 weeks of age). Although, of interest from an islet-regeneration perspective, the age-specific decrease in proliferation was also observed in healthy wild-type mice following their last phase of growth. A decrease in proliferation was promptly followed (between 6 and 8 weeks of age) by the strong emergence of an immune system and inflammatory signature, which dominated the transcriptional landscape, confirming the findings of previous studies^21^. This signature was further reinforced in the next period (between 8 and 10 weeks of age), but largely reached a regulatory plateau in the last period analyzed (between 10 and 12 weeks of age).

Specifically, our analysis showed that the inflammatory signature was organically centered around key immunogenic factors (*Il1b, Tnf, Infg,* and *Ccl2*) as early as its emergence (between 6 and 8 weeks of age). The establishment of this proinflammatory signature before any evident signs of immune cells infiltration, suggested that islet endocrine cells might play a role in the initiation of the early events leading to insulitis. However, this issue cannot be properly settled with the current experimental design and thus it is still unclear what cell population is responsible for the early proinflammatory signature.

Moreover, the upregulation of the immunogenic factors was also mirrored by Atf3 (one of their main regulators) and by Hh-signaling pathway. Previous studies pinpointed *Atf3* as a stress-induced transcription factor in a multitude of tissues and cell types. It acts as a critical hub in regulating metabolism and immunity, by integrating a broad range of signals, such as chemokines or endoplasmic reticulum stress, amongst others^30,31^. Although Atf3 drives an anti-inflammatory network in most tissues^32–36^, previous studies have demonstrated its proinflammatory role in the pancreatic islets by driving the expression of *IL1B, IL6, TNF* and *CCL2*^25^ following stress stimulation caused by hypoxia and transplantation. Here, we propose a potential involvement of Atf3 in a proinflammatory loop that spirals towards insulitis in the NOD mice. However, further studies are required to comprehensively validate and characterize this issue.

The Hh-signaling inactivation during pancreas development is absolutely required for pancreas organogenesis^37–40^ and its modulation also plays a role in adult pancreas regeneration, being involved in regenerative cell conversion events^41^. Here, we observed the deregulation of key members of the Hh-signaling pathway and wanted to further investigate its potential relationship with Atf3 regulation. Unfortunately, our pilot experiment failed to establish a clear connection between the Hh-signaling pathway and ATF3 regulation, probably because of a large variation in number of ATF3 positive cells per sc-islet. A tendency toward both a decrease in the ATF3 signal intensity and in the number of ATF3-expressing cells was observed following Hh-signaling activation, suggesting the Hh-signaling as a negative feed-back loop of ATF3 regulation, at least in this very simplified in vitro system. However, as this difference was not statistically significant, additional experiments are absolutely required for the characterization of Hh-signaling role in islet stress and inflammation. It should also be considered that the absence of Hh-signaling impact in human sc-islets does not exclude a role for the Hh-pathway in Atf3 regulation, in vivo, in mice or the occurrence of a Hh pathway-Atf3 regulatory loop in immune cells. Settling these issues will require different and more complex methodological approaches such as single-cell RNA sequencing.

One limitation of the current study design is the impossibility of demultiplexing the observed signatures according to the cell type where they are expressed. Consequently, by using bulk RNAseq on isolated islets one cannot distinguish between the resident islet cells and infiltrating immune cells. Moreover, the regulation of Hh-signaling and Atf3 could occur in different cell types, thus their opposing activity patterns might represent a case of intercellular signaling instead of an intracellular regulatory loop.

Finally, one of the main drawbacks of this study is the insufficient experimental validation of the transcriptional landscapes described. However, in this short report, we intended to simply revisit and characterize the global molecular events leading to diabetes in the NOD mice by using the increased power of the next-generation sequencing and pathway analysis tools, with the goal of unveiling new regulators and molecular links underlying future experimental studies.

## Materials and methods

### Mice

To study the onset of insulitis in mouse islets, we used the NOD/ShiLtj mouse model (RRID:IMSR JAX stock #001976, referred to as NOD^42^), in which mice develop diabetes as a result of autoimmune attack^19^. Animals were housed in a specific pathogen-free animal facility. They were maintained on a 12 h light and 12 h dark cycle and had ad libitum access to food and water. All the experimental procedures involving animals were conducted in accordance with the European Union (EU) Directive 2010/63/EU and approved by the national competent authority (Authorization No. 590/13.01.2021). The study is reported in accordance with ARRIVE guidelines.

For this study, NOD female mice of reported ages were used, as described in the Results section and figure legends.

### Islet isolation

Islets were isolated from NOD female mice of 4-, 6-, 8-, 10- and 12-weeks old (n = 3 mice per each time point) using a protocol described in Daian et al*.*^43^, with minor changes. Briefly, the mice were euthanized by cervical dislocation, and the pancreas was perfused with a solution of 0.5 mg/ml collagenase XI (Sigma-Aldrich, St. Louis, MO, USA; #C7657) and 0.8 mg/ml bovine serum albumin (BSA, Sigma-Aldrich; #A2153) dissolved in Hanks’ balanced salt solution (HBSS) supplemented with Ca^2+^ and Mg^2+^ (Carl Roth, Karlsruhe, Germany; #9119.1), via hepatopancreatic duct cannulation. The pancreas was harvested, transferred to a tube with collagenase solution and incubated in a water bath for 15 min, at 37°C. The digestion was stopped by adding cold RPMI medium (Corning, NY, USA; #10-040-CV) supplemented with 10% FBS (PAN-Biotech, Aidenbach, Germany; #P30-3306). After mechanical dispersion by energic shaking, the pancreas was passed through a metallic strainer and washed two times with RPMI. Islet separation was performed on a gradient made of 5ml of Histopaque-1119 (Sigma-Aldrich—#11191), overlaid with 5 ml of Histopaque-1077 (Sigma-Aldrich—#10771) and topped with 5 ml RPMI. The gradient was centrifuged at 850×*g* for 15 min, at room temperature. The islets were transferred from the gradient to a new tube and washed three times with RPMI, then they were resuspended in RPMI with 10% FBS, transferred to a nonadherent dish and hand-picked. The obtained islets were lysed in RLT buffer (Qiagen, Redwood City, CA, USA-#79216) and stored at − 80 °C.

### RNA extraction

RNA was isolated from the lysed islets using an RNeasy Mini Kit (Qiagen, Redwood City, CA, USA; #74104), according to the manufacturer’s instructions. In the final step, RNA was eluted from the columns with 20 μl of ultrapure water and the concentration was determined using a NanoDrop 2000c (ThermoFisher Scientific, Waltham, MA, USA).

### Sequencing

Total RNA samples were shipped to Novogene (Cambridge, UK), where a second quality control and library preparation (polyA enrichment) were performed. Sequencing was performed on an Illumina Novaseq PE150 platform—30 million reads each end for a total of 9G raw data output.

### Data and pathway analysis

Files from sequencing were processed in the CLC Genomics Workbench 23.0 (Qiagen, Aarhus, Denmark). Pre-processing included adapter and quality score-based trimming, using the default setting provided by the trimming tool in the CLC software. Alignment and quantification were performed using the RNAseq Analysis tool, also adhering to the default settings chosen by the CLC Workbench. To generate the DEG lists, groups were compared using the “Empirical Analysis of DGE” algorithm of the CLC software. The DEG lists were subsequently uploaded to Ingenuity Pathway Analysis for further analysis^44^ as previously described^45,46^. Only DEGs that met a threshold FC > 1.5, and *p* < 0.05 were used for pathway prediction, further Network settings were kept at the default. All data sources were included but limited to a mouse origin and experimentally validated observations.

### Glycaemia measurements

Blood glucose levels were measured weekly via puncture of the lateral tail vein and using an Accu-Chek Performa glucometer (Roche, Basel, Switzerland) with the corresponding strips. Between 3 to 15 female mice were analyzed at 4-, 6-, 8-, 10-, 12- and 28-weeks old. Mice were considered diabetic after they had two consecutive blood glucose readings exceeding 200 mg/dL (11 mmol/L) on two consecutive days.

### Pancreas collection and immunostaining

Harvested pancreata from at least 3 NOD female mice per reported age timepoint were fixed in 4% PFA (Roth), for 2h at 4 °C, dehydrated on a sucrose gradient for 1.5 h, embedded in optimum cutting tissue medium (CellPath), frozen and stored at − 80 °C. Cryosections with a 5 µm thickness were collected on slides, rehydrated in PBS, followed by 20 min of permeabilization with 0.5% Triton X-100 and a 30 min incubation with 1% BSA blocking solution at room temperature. Incubation with primary antibody, diluted in 1% BSA, for 1 h at room temperature was conducted using the following antibodies: monoclonal rabbit anti-Insulin (ThermoFisher Scientific, Waltham, MA, USA, 701265, 1:1000), monoclonal mouse anti-Glucagon (Novus Biologicals, NB600-1506, 1:500), polyclonal rabbit anti-Ki67 (ThermoFisher Scientific, Waltham, MA, USA, MA5-14520, 1:400), polyclonal rabbit anti-Atf3 (Sigma Aldrich, HPA001562, 1:100), monoclonal mouse anti-CD45-AlexaFluor647 (BioLegend, 160304, 1:50). Secondary antibodies were from Invitrogen and were used at 1:200 dilution. They were incubated for 45 min at room temperature, followed by DAPI counterstaining of cell nuclei and mounting with antifade mounting media (Vector Laboratories, Burlingame, CA, USA, H-1000).

### Microscopy and analysis of mouse pancreas sections

Images of labelled mouse islets were acquired with a fluorescence microscope Leica DMi8 (Leica Microsystems, Wetzlar, Germany), in the same imaging session for each experiment, and were processed using LasX software (version 3.7.6.25997), using the same parameters for each channel, for every experimental set. Image analysis was conducted using QuPath software (10.1038/s41598-017-17204-5, version 4.0), as a quantification tool, on raw images. This software allows associations of three different fluorescence channels, blue, green, and red, for quantification. Thus, the following sets were proposed for analysis: insulin and glucagon, insulin and Atf3, and insulin and Ki67, while DAPI (blue) was present in each of them. For that, the pseudo-colors of the images were post-acquiring-modified accordingly. Cell detection was carried out based on nuclei staining with DAPI, by adjusting the intensity threshold to capture the majority of the cells in the region of interest, defined manually around the pancreatic islet. The cytoplasmic area of each detected cell was defined by adjusting the cell expansion feature available in the QuPath and was kept constant throughout all the quantifications. The mean fluorescence intensities for the proteins of interest (insulin, glucagon, Atf3), detected in the previously defined cytoplasmic, or nuclear (Ki67, Atf3) areas of the cells, were measured by setting the suitable threshold for each channel.

### Cell source and maintenance

Commercially available human induced pluripotent stem cells (hiPSC), generated by retroviral reprogramming of PGP1 donor skin fibroblasts (Coriell, GM23338), were maintained as previously described^26,39^ in mTeSR Plus cGMP stabilized feeder-free maintenance medium (Stem Cell Technologies, 100-0276). The passaging of hiPSCs was performed by fragmenting the existing colonies after incubation with Gentle Cell Dissociation Reagent (StemCell technologies, 100-0485). The hiPSC cultures were negative for mycoplasma tested by MycoAlert Mycoplasm Detection Kit (Lonza, LT07-418) prior to induction of differentiation.

### In vitro differentiation and GLI modulators

The hiPSCs were differentiated towards pancreatic islet-like clusters following a previously published stepwise protocol^26,27^ starting with 1,500,000 cells/well in 6-well plates pre-coated with Geltrex LDEV-Free Reduced Growth Factor (Gibco, A1413202). For GLI inhibition, sc-islets were treated with 10 µM GANT58 (Sigma-Aldrich, G8923) or 10 µM of Purmorphamine added to the stage 7 medium for 24 h followed by fixation and processing.

### Immunofluorescence of sc-islets

Collected sc-islets were fixed in 4% PFA, prior to dehydration in a sucrose gradient and embedding in Tissue Tek OCT (Sakura). Frozen blocks were cut into 5µm sections that were used for staining. Sections were rehydrated in PBS, prior to permeabilization (15 min, 2% Tritonx-100 in PBS) and blocking (30 min, 2% BSA in PBS). The primary antibodies used were polyclonal rabbit anti-ATF3 (1/100, Sigma Aldrich, HPA001562), rat anti-C-peptide IgG2a (1/250 DSHB, GN-ID4), and guinea pig IgG anti-glucagon (1/400 ABCD antibodies, ABCD-TA011). Following brief PBS washes, the sections were incubated for 1 h at room temperature in the dark, with donkey anti-rabbit A647 (1/500, Molecular Probes, A-31573), goat anti-rat A594 (1/500, Molecular Probes, A-11007), goat anti-guinea pig A488 (1/500, Molecular Probes, A-11073) and DAPI nuclear staining (D1306, Molecular Probes). Finally, glass slides were mounted on to the sections using Aqueous Mounting Medium (ab128982, Abcam).

### Sc-islets imaging and image analysis

Stained slides were imaged on a confocal microscope, Leica SP8 STED, to produce high resolution images for quantification of protein expression and staining intensity. Quantification was performed either manually or performed by using the FIJI (ImageJ 2.9.0) software for evaluation of stain intensity and supervised automated counting. The intensity of ATF3 expression was measured using FIJI (ImageJ 2 V2.14.0/1.54f). Before conducting intensity measurements, the threshold was set to 45, identified as the background signal intensity. FIJI then calculated the mean grey value of ATF3 signal within the individual stem cell islets.

For the automated supervised counting, a macro using Auto Threshold feature with the Otsu Dark method in FIJI was used. A region of interest (ROI) was drawn manually to define the islet area from the generated mask before cells were counted by “Automatic Particle counting” with a size limit set at 10µm.

### Statistics

Graphs and statistics were done in GraphPad Prism, version 9.5.1 (GraphPad Software, Boston, MA, USA).

### Supplementary Information

Supplementary Figure 1.

Supplementary Table 1.

## Data Availability

The full datasets were deposited to the NCBI Gene Expression Omnibus Repository^47^, accession number GSE256150. Data sets are at https://www.ncbi.nlm.nih.gov/geo/query/acc.cgi?acc=GSE256150
