# Supplementary material for: Molecular profiling of NOD mouse islets reveals a novel regulator of insulitis onset
Source: Sci Rep. 2024 Jun 25;14:14669. doi: 10.1038/s41598-024-65454-x (PMC11199597; doi:10.1038/s41598-024-65454-x)
Supplement: Supplementary file 1 — Supplementary Figure 1. [file 41598_2024_65454_MOESM1_ESM.pdf]

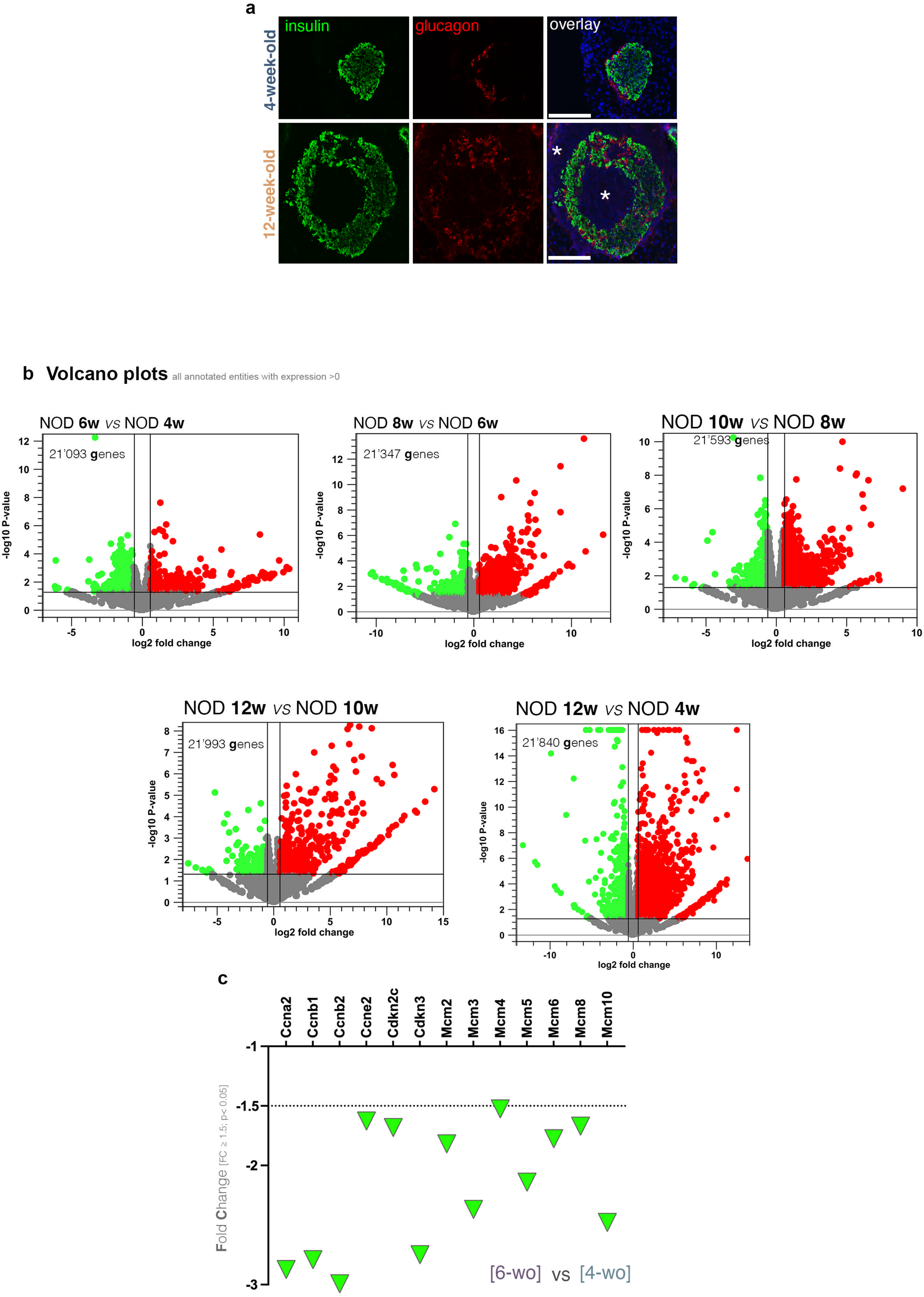
a
insulin
glucagon
overlay
4-week-old
12-week-old
b Volcano plots all annotated entities with expression >0
NOD 6w vs NOD 4w
21'093 genes
NOD 8w vs NOD 6w
21'347 genes
NOD 10w vs NOD 8w
21'593 genes
NOD 12w vs NOD 10w
21'993 genes
NOD 12w vs NOD 4w
21'840 genes
-log10 P-value
log2 fold change
c
Ccna2
Ccnb1
Ccnb2
Ccne2
Cdkn2c
Cdkn3
Mcm2
Mcm3
Mcm4
Mcm5
Mcm6
Mcm8
Mcm10
Fold Change [FC ≥ 1.5; p< 0.05]
[6-wo] vs [4-wo]

SUPPLEMENTAL FIGURE LEGEND

Supplemental Figure 1 (a) Representative immunofluorescence images of insulin (green), glucagon (red) and DAPI staining (blue) at 4-weeks and 12-weeks of age in NOD mice, with asterisk pinpointing insulitis (scale 100 µm). **(b)** Volcano plots and total number of genes annotated of each comparison **(c)** Graph displaying the observed statistically significant downregulation of key proliferation markers in the RNAseq dataset ($FC \geq 1.5$, $p < 0.05$) between 6-weeks and 4-weeks of age in NOD mice.
